# Report from an online workshop on family physician socialization: Reaffirmation using a hypothetical case

**DOI:** 10.1002/jgf2.587

**Published:** 2022-10-13

**Authors:** Shohei Taniguchi, Lee Young, Tsubasa Nakai, Kazuoki Inoue

**Affiliations:** ^1^ Department of Internal Medicine Nichinan Town National Health Insurance Nichinan Hospital Tottori Japan; ^2^ Department of Community‐Based Family Medicine, School of Medicine Tottori University Faculty of Medicine Tottori Japan; ^3^ Department of Internal Medicine Hino Hospital Tottori Japan; ^4^ National Health Insurance Daisen Clinic Tottori Japan

## Abstract

This letter provides a report of our online workshop on the professional socialization of family physicians attended by family medicine residents and staff doctors in Japan. Participants engaged in small group discussions after reading hypothetical cases that threatened the identity of family physicians. Participants considered the importance of reaffirming and improving their strengths during discussions.

To the Editor,

Healthcare workers are socialized to be professionals. Professional socialization is the process of internalizing the specific culture of a professional community, including expectations, values, beliefs, customs, traditions, responsibilities, and unwritten rules of the profession, such as understanding the hierarchy. During this process, professional identity is formed, and professional competence is acquired.^1^ On the contrary, it has been reported that medical students develop a lack of empathy and increasingly focus on biomedical aspects, which may be an effect of socialization, as they progress through medical education.^2^ While socialization is an important aspect of professional development, it is important to note that medical practitioners may be unconsciously influenced by socialization. However, unlike hospital specialists, such as neurosurgeons and gynecologists, family physicians often have difficulty in appreciating the value and role of their specialty. Because, the socialization of family physicians is influenced by the context of their workplace.^3^

In February 2022, we held an online workshop at the Family Medicine Winter Seminar for Young Doctors entitled, “Thinking about Taking Family Medicine for granted,” in Japan. A total of 20 participants (13 residents and seven staff grade doctors) attended the workshop. The purpose of the workshop was to increase awareness through a dialogue about the role of family physicians and the effects of socialization. Identity is formed unconsciously through socialization. We believed that participants become conscious of their socialized selves by becoming conscious of their identities. We have prepared a hypothetical case based on the assumption that identity is made conscious when a situation in which the identity of a family physician is threatened. Participants read the hypothetical case and discussed three questions in small groups of three to four participants (Table 1). Finally, participants were encouraged to reflect throughout the discussion.

**TABLE 1:** Hypothetical case and questions in the workshop

Hypothetical case
An AI diagnostic tool has been developed in 2040 that has 98% diagnostic accuracy using high‐performance ultrasound and low‐volume blood tests. The AI is able to create explanatory models based on patient statements and facial expressions during patient consultations. This tool has been introduced at the hospital where you work as a family physician and has been well‐received by patients. Some patients have suggested that AI should be integrated into all medical departments
Questions
① What did you feel when you read the case? ② Referring to the seven qualities and abilities (competencies) of family physicians,^4^ consider the strengths of family physicians in 2040 as the use of AI increases ③ What contribution can the Department of Family Medicine make to this hospital? Summarize the points you would discuss with the hospital director

During group work, many groups considered integrative care and patient‐centered medicine as strengths of family physicians. Many groups stated that they believed family physicians can handle highly individualized and complex cases with the use of AI. Many participants felt that the raison d'etre of family physicians would be threatened in the scenario. However, participants considered the importance of reaffirming and improving their strengths as the discussion proceeded.

All participants completed a postevaluation form. A total of 16 participants responded to the question: “How can you apply what you learned in this session to your fieldwork?” Responses were categorized into two themes. The first theme involved communication of the role of family physicians to others. The second theme involved appreciation and development of the strengths of family physicians.

This workshop presented participants with a hypothetical case in which both their own specialty and their role were threatened to help participants become conscious of their socialized selves by becoming conscious of their identities. Discussions around this scenario led to a reaffirmation of the role of family physicians; however, the effect of these discussions on clinical practice is unknown. Furthermore, the negative effects of family physician socialization were not discussed and will be the subject of future research.

## CONFLICT OF INTEREST

The authors declare no conflicts of interest for this article.
